# Is wear debris responsible for failure in alumina-on-alumina implants? Clinical, histological, and laboratory investigations of 30 revision cases with a median follow-up time of 8 years

**DOI:** 10.3109/17453670902876730

**Published:** 2009-04-01

**Authors:** Lucia Savarino, Nicola Baldini, Gabriela Ciapetti, Andrea Pellacani, Armando Giunti

**Affiliations:** ^1^Laboratory for Pathophysiology of Orthopaedic ImplantsBolognaItaly; ^2^Division VII Istituti Ortopedici RizzoliBolognaItaly

## Abstract

**Background and purpose** Ceramic-on-ceramic articulation is an attractive alternative to metal-on-polyethylene (PE) bearings, but little is known about the in vivo effects induced by dissemination of alumina wear debris in the periprosthetic tissues. We hypothesized that wear debris is not the main factor responsible for loosening and failure of the implant but that mechanical problems caused by incorrect surgical technique, prosthetic design, or trauma, may cause instability of the implants and result in production of wear debris.

**Patients and methods** Clinical, radiographic, laboratory, and microbiological data from 30 consecutive patients with failed alumina-on-alumina arthroplasties, 19 with screwed socket and 11 with press-fit socket, were systematically collected and evaluated. Retrieved peri-implant tissues and prosthesis wear were also analyzed.

**Results and Interpretation** Loosening was due to malpositioning, primary mechanical instability, trauma, or infection. Bone stock was generally preserved, even if screwed implants showed higher levels of osteolysis. Variable implant wear and tissue macrophage reaction were present but activation of giant cells/osteoclasts was not induced, and no correlation between histocytic reaction and the level of osteolysis was found. These findings indicate that, in contrast to the situation with metal-on-PE bearings, wear debris and occasional osteolysis were the effect rather than the cause of failure of ceramic-on-ceramic implants, and that press-fit socket fixation was the socket fixation design of preference.

## Introduction

Much effort has been made to develop alternative highly wear-resistant materials for bearing surfaces (such as metal-on-metal and ceramic-on-ceramic) in order to eliminate or reduce wear and particle-induced osteolysis at the implant-bone interface, which can lead to macrophage- and osteoclast-induced bone resorption, implant loosening, and failure (Goodman et al. 1998, Schmalzried and Callaghan 1999, Archibeck et al. 2001, Jacobs et al. 2001, Bauer 2002). Ceramic-on-ceramic articulation, introduced by Boutin in France in 1970 (Sedel 2000), provides low friction and wear, a high Young’s modulus, high resistance to compression, extreme hardness, and biocompatibility; moreover, it has been shown to be non-deformable under shock, heat, or pressure (Sedel 1997). The quantities of wear particles in the periprosthetic tissues around alumina-on-alumina bearings have been found to be 2–22 times lower than those observed around metal-on-PE articulations (Böhler et al. 2000). Despite the excellent wear performance, however, the clinical results have generally been less satisfactory than those of established metal-on-PE designs; the quality of the first generation of alumina was not optimal, with a large grain size, a wide distribution of grain-size and low purity (Böehler et al. 1994, Sedel 1997, Hamadouche et al. 2002). In recent years, better results have been reported due to improvements in the material manufacturing process, which has led to the production of a new generation of surgical-grade dense alumina ceramic (Sedel et al. 1994, Huo et al. 1996, Bos and Garino 2000, Willmann 2001). Even so, other researchers have reported severe wear from loosened ceramic-on-ceramic prostheses retrieved at revision surgery (Nevelos et al. 1993, Böehler et al. 1994). In addition, little is known about the accumulation of alumina wear debris in periprosthetic tissues and its in vivo effects (Yamamoto et al. 2005).

We hypothesized that wear debris may not be responsible for the loosening and failure of ceramic-on-ceramic implants but, instead, that mechanical problems may cause instability of the implants and result in production of wear debris. We evaluated a consecutive series of failed alumina-on-alumina total hip arthroplasties at a median follow-up time of 8 years, and investigated the occurrence of bearing wear and wear-associated osteolysis. Clinical and laboratory data were examined in addition to retrieved peri-implant tissues.

## Patients and methods

We enrolled a consecutive series of 30 patients with failed alumina-on-alumina THAs. Patients had had a unilateral implant for the treatment of primary osteoarthritis (OA) or secondary to developmental hip dysplasia (DHD), trauma, coxititis, or necrosis. The AnCA ceramic bearing (Cremascoli Ortho Sp.A., Milan, Italy) had been used in 19 cases: an old generation of alumina head 32 mm in diameter (Biolox, Cerasiv GmbH, Plochingen, Germany), and a dense alumina socket, contoured with a threaded titanium alloy ring, and coated on its external surface with a 3D porous alumina. Cremascoli Ortho manufactured the CoCr alloy stem, 13 cm long and anatomically shaped with a porous hydroxyapatite (HA) coating at the proximal part. The AnCA Fit acetabular cup (Cremascoli Ortho) had been used in 11 THAs: it was a hemispheric titanium-made cup, torsionally stabilized by 2 bladed pegs. The outer surface was coated with 2 layers of titanium beads and the internal part had a truncated tapered cone fitted with a suitable ceramic liner of the new generation (Biolox Forte; Ceramtec, Plochingen, Germany). This articulated against a 28-mm diameter ceramic head (Biolox; Ceramtec). A TiAl6V4 alloy constituted the cementless femoral component: 12 cm long, anatomically shaped, with grid blasted surface, grooves in the proximal half and HA-coated (Cremascoli Ortho) (Tables 1 and 2). At the time of retrieval, no patient had been involved in strenuous activities.

**Table 1.** Characteristics of the patients

Sex: men/women	11/19
Age at the time of implantation^a^	56 [9] (31–74)
Diagnosis	
Primary osteoarthritis	16
Secondary osteoarthritis	
DHD	5
trauma	2
coxitis	1
femoral stem fracture	1
osteonecrosis	5
Age at the time of revision^a^	65 [10] (39–86)
Follow-up (years) **^a^**	8 [8] (0.4–17)

^a^Mean [SD] and (range)

**Table 2.** Patient demographics and characteristics of implants

Case	Sex	Age (years)	Etiology	Primary surgery	Follow-up (years)	Socket type	Ceramic quality
1	F	71	POA	Yes	12.4	S	Biolox
2	M	65	POA	Yes	13.0	S	Biolox
3	M	57	PTN	Yes	9.6	S	Biolox
4	M	67	POA	Yes	5.1	P	Biolox F
5	F	66	POA	Yes	13.0	S	Biolox
6	F	68	POA	Yes	14.3	S	Biolox
7	M	55	PTN	Yes	1.4	P	Biolox F
8	F	68	Trauma	No	0.7	P	Biolox F
9	F	68	PTN	Yes	4.0	S	Biolox
10	M	67	POA	Yes	6.8	S	Biolox
11	M	71	POA	Yes	9.8	S	Biolox
12	F	65	POA	Yes	3.6	S	Biolox
13	F	86	POA	Yes	17.0	S	Biolox
14	F	70	DHD	Yes	3.8	P	Biolox F
15	F	70	Fracture	Yes	7.8	P	Biolox F
16	F	60	Coxitis	Yes	10.1	S	Biolox
17	M	39	SOA	Yes	9.0	S	Biolox
18	M	70	PTN	No	10.0	S	Biolox
19	F	54	POA	Yes	1.6	P	Biolox F
20	F	80	DHD	Yes	5.4	P	Biolox F
21	F	71	POA	Yes	12.0	S	Biolox
22	F	55	DHD	Yes	1.3	P	Biolox F
23	F	69	POA	Yes	13.0	S	Biolox
24	F	66	POA	Yes	8.6	S	Biolox
25	M	55	POA	Yes	9.6	S	Biolox
26	M	67	POA	Yes	1.2	P	Biolox F
27	M	76	AVN	Yes	14.1	S	Biolox
28	F	48	DHD	Yes	2.9	P	Biolox F
29	F	76	POA	Yes	10.9	S	Biolox
30	F	44	DHD	Yes	0.4	P	Biolox F

POA: primary osteoarthritis;

SOA: secondary osteoarthritis post trauma;

DHD: developmental hip dysplasia;

PTN: posttraumatic necrosis;

AVN: avascular necrosis;

S: screw-fit; P: press-fit.

A standard radiograph of the pelvis and a lateral one of the hip were taken in all patients and the presence of osteolysis was defined (Gruen et al. 1979). Gruen zone classification around the stem and DeLee and Charnley classification around the cup (DeLee and Charnley 1976) were used. An osteolysis score of 0 to 3 was given, with 0 meaning absence of osteolysis, 1 meaning radiolucent lines < 2 mm, 2 meaning focal osteolysis or radiolucent lines enclosed between 2 and 10 mm, and 3 meaning focal osteolysis or radiolucent lines of > 10 mm.

Total body bone scan with technetium 99m-labeled hydroxymethylene diphosphonate [(99m)Tc-HDP] was performed in 20 of the 30 cases in order to evaluate prosthesis loosening. Technetium-99m hexamethyl propyleneamineoxide [(99m)Tc-HMPAO]-labeled granulocyte scintigraphy was carried out in 18 of the 30 patients to detect periprosthetic infection (Sudanese et al. 1994). Microbiological analysis was performed in 10 patients (Table 3; see supplementary data).

White blood cell (WBC) count, erythrocyte sedimentation rate (ESR, mm/h), C-reactive protein (CRP, mg/dL), and plasma fibrinogen (mg/dL) were determined in all patients.

**Figure 1.**
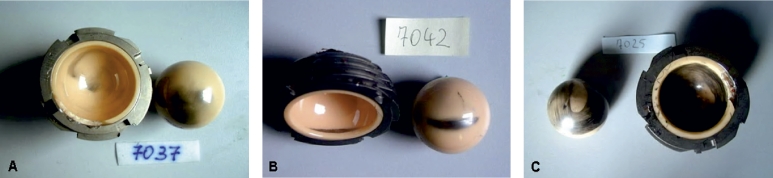
Wear in explanted ceramic components: superficial (A; case 16), striped (B; case 2), and massive with deformity (C; case 25).

As part of the revision arthroplasty procedure, implants were removed and tissue biopsies retrieved. 3 standardized bioptic sites were analyzed in total revisions, i.e. newly-formed joint capsule, reactive tissue between the prosthetic stem and the femur, and reactive tissue between the acetabular component and the iliac bone. In partially removed implants, newly formed joint capsule and reactive tissue adjacent to the prosthetic stem or to the socket were examined, depending on the retrieved component. Paraffin-embedded tissues were sectioned and hematoxylin-eosin stained. Two evaluators without any knowledge of patient data performed the histopathology analysis. The type and degree of peri-implant inflammation was defined by a scoring system (0–3) (Pizzoferrato et al. 1988). A semiquantitative evaluation of the abrasion rates of the explanted ceramic parts was also performed, using a hard pointer on the tip of a stylus that touched the surface of the component and was driven over the surface (Figure 1). The abrasion rate was classified as non-existent, superficial, striped, or massive with macroscopic deformity of the coupling components, and was compared to the degree of inflammatory reaction.

### Statistics

Fisher’s exact test was used to assess a possible correlation between grade of osteolysis and wear/histiocytic reaction, and the effects of socket design on the same parameters. Specific differences concerning laboratory parameters were analyzed with the Mann-Whitney U test. All statistical analyses were performed using StatView 5.01 for Windows (SAS Institute Inc., Cary, NC).

### Ethics

The clinical study was approved by our institutional ethics committee on human research (ISTO, Prot. 6 CE/US/ml) and was performed in compliance with the Helsinki Declaration (2004).

## Results

Preliminary statistical analysis showed that sex and age of the subjects with different kinds of socket fixation would not introduce any significant differences in the variables.

Indication for revision surgery, as judged from the combination of clinical, radiographic, and microbiological data, was mechanical instability in 21 cases, idiopathic loosening in 2 cases, evidence of infection in 6 cases, and breakage in 1 case (Table 3; see supplementary data). Cases of infection were evaluated separately.

**Figure 2.**
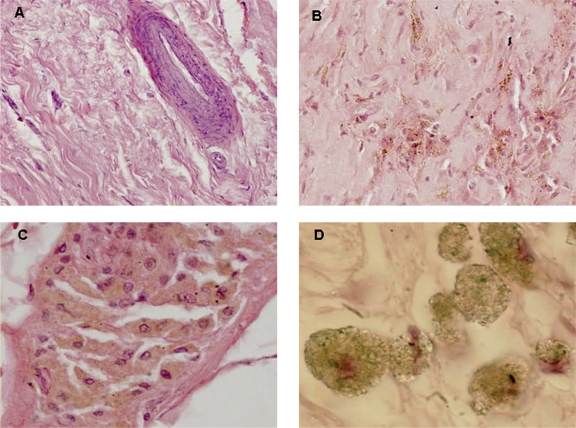
Morphological evaluation of periprosthetic biopsies. A. Minimal debris and no significant reaction; H and E staining, ×10. B. Significant debris mostly within macrophages; H and E staining, ×20. C. Massive debris and extensive reaction; H and E staining, ×10. D. Macrophages filled with alumina particles at higher magnification; H and E staining, ×60.

16 of the 21 cases with mechanical failure had a screwed socket while 5 cases had the press-fit fixation. Tissue wear and the osteolysis grade are given in Table 4 (see supplementary data). Periprosthetic tissues showed a mixed pathology with areas of normal tissue, areas that were relatively rich in macrophages with abundant cytoplasm and little necrosis, scar-like fibrosis, and micro-hemorrhages (Figure 2). The small granular wear particles were usually present as agglomerates phagocytosed in the cytoplasm of macrophages, or in distinct channels in the tissues.

A correlation was found between the amount of wear and the grade of histiocytic reaction (p = 0.02), but there were no statistically significant effects (Fisher’s exact test) induced by the presence of tissue wear particles and histocytic reaction on the osteolysis process (p = 0.2 and p = 0.3, respectively).

The same test was used in order to evaluate the effect of socket fixation (screwed vs. press-fit) in implants retrieved for aseptic loosening: they did not show any statistically significant differences concerning histiocyte reaction (p = 0.7), wear debris (p = 0.4), or osteolysis (p = 0.09).

The abrasion rate was non-existent in 8 cases, superficial in 7, striped in 10, and massive with macroscopic deformity of the components in 5 cases (Table 4; see supplementary data). There were no statistically significant differences in abrasion rate in patients with different types of socket (p = 0.29, Fisher’s exact test). In addition, no correlation was found between the cell reaction and the abrasion rate, or between the grade of osteolysis and the abrasion rate.

A difference was found between septic and aseptic cases concerning CRP and fibrinogen levels (p = 0.01 and 0.02, respectively; Mann-Whitney U test) whereas this was not the case for ESR and WBC counts (p = 0.4 and 0.08, respectively) (Table 5). Similarly, no statistically significant differences in C-RP (p = 1.0), ESR (p = 0.7) and fibrinogen (p = 0.7) were found between screw-fit and press-fit implants with aseptic failure.

**Table 5.** White blood cell numbers, erythrocyte sedimentation rate (ESR) expressed as mm/h, C-reactive protein (CRP) expressed as mg/dL, and plasma fibrinogen (mg/dL), reported as mean with standard error (SE), median value, and range

Index	Normal values	Aseptic loosening				Septic loosening			
mean	[SE]	median	(range)	mean	[SE]	median	(range)
ESR (mm/h)	≤ 15	29	[2.8]	26	(7–53)	43	[15]	35	(14–102)
CRP (mg/dL)	< 0.5	0.5	[0.1]	0.6	(0.01–1.8)	2.1	[0.95]	1.2	(1.1–4.0)
White blood cells (× 10^3^ cells/mm^3^)	4.5–9.5	5.6	[0.3]	5.0	(4.9–9.4)	8.0	[1.4]	7.8	(4.6–13.9)
Fibrinogen (mg/dL)	150–400	363	[12]	367	(292–424)	502	[34]	486	(406–618)

The presence of neutrophils was detected in all cases with a clear diagnosis of infection except 1, and a correlation was found between the presence of neutrophils and a positive microbiological result (p = 0.03). On the other hand, there was no statistically significant correlation between the presence of lymphocytes and positive culture (p = 0.8), and none between the presence of plasma cells and positive culture (p = 0.2, Fisher’s exact test). Scintigraphy with [(99m)Tc-HMPAO]-labeled granulocytes did not reveal infection in 3 of these cases.

## Discussion

The tribologic and biological properties of recent bearing materials for THA have been well studied (Revell et al. 1997, Kadoya et al. 1998, Campbell et al. 2004). New-generation ceramic-on-ceramic couplings appear to offer a good alternative, due to the low rate of wear and satisfactory clinical results (Bizot et al. 2001). However, the pathogenesis of failure in patients with ceramic-on-ceramic couplings has not been well documented.

We hypothesized that wear debris is not the main cause of loosening and failure of such implants. Instead, mechanical problems might cause instability of the implants, resulting in production of wear debris. Our hypothesis is in line the results of Lusty et al. (2007) who studied 33 ceramic-on-ceramic bearings in retrievals from THA and showed that in vivo wear volumes are small in comparison to other bearing materials. Also, we consider that analysis of retrieved specimens is essential for an understanding of the pattern and the true magnitude of wear. Consequently, we evaluated 30 ceramic-on-ceramic failures from a total original ceramic-on-ceramic implant series covering a period that involved about 3,000 surgeries. Clinical and radiographic evaluation, laboratory tests, and histopathology showed that failure was due to infection in 6 patients and that aseptic loosening occurred in the remaining 24 patients.

One limitation of our histological study was the small number of bioptic sites used for the analysis and the heterogeneity of peri-implant tissues; thus, the limited sampling may not have been representative. Another limitation is that material from only 10 patients underwent culture; in the other cases, the clinicians did not deem it necessary to perform microbiological analysis.

A few histological studies of retrieved periprosthetic tissues have been performed, but they have reported contradictory findings. This may have been the result of different designs and compositions of the implants, as well as on different types of coupling (Henssge et al. 1994, Lerouge et al. 1997, Sedel 1997, Fenollosa et al. 2000, Bizot et al. 2001, Hatton et al. 2002). Concerning osteolysis, some authors have shown that a low volume of debris is generated from well-functioning ceramic-on-ceramic bearings, either of the old (Hamadouche et al. 2002) or of the new generation (Fenollosa et al. 2000), and the material seemed unlikely to produce an osteolytic response. In contrast, Yoon et al. (1998) reported a high incidence of osteolysis in patients with uncemented prostheses, but an older generation of ceramic was used. They described an interfacial connective tissue rich in macrophages, with large amounts of ceramic particles, and concluded that ceramic wear could stimulate a foreign body response leading to periprosthetic osteolysis.

In the present study, all the aseptic cases showed a mechanical cause of failure—recognized as malpositioning, trauma, or primary mechanical instability. The high rate of mechanical failure in the patients with a screw socket was mainly related to the improper design of the socket itself. Micromotion associated with the screw fixation probably induced vascular damage and higher levels of osteolysis, in contrast to the implants with a press-fit socket where the bone stock was generally well preserved. In some cases, incorrect socket positioning or incorrect stem size induced micromovements, and eventually loosening.

Retrieved ceramic components always showed some degree of wear, and variable amounts of particles were present within macrophages. It is noteworthy that no significant effect was induced by the presence of particles and histocytic reaction on the osteolysis process. We ascribe this to the absence of polyethylene wear, which mainly gives larger particles (8–15 µm) than found for ceramic materials, with substantial biological effects that include an extensive degree of foreign-body reaction. This is also supported by our own previous findings. We challenged macrophages with alumina and PE particles isolated from tissue fragments obtained at revision arthroplasty (Mochida et al. 2001), and performed a cell culture medium assay of PGE2, an inflammatory mediator responsible for macrophage and pre-osteoclast recruitment and differentiation. The assay showed that there was a high degree of release induced by PE particles (150–160 ng/mL) whereas alumina caused only slight modification of the basal level (40–65 ng/mL vs. 30 ng/mL). In another study, in order to highlight the differences between Al2O3 and PE particles we investigated osteoblast-osteoclast interaction in the presence of these materials. Our data showed that ceramic wear debris had less ability to influence osteoblast-osteoclast cooperation and to induce osteoclast formation than PE (Granchi et al. 2004). The present study confirms our previous observations and shows that even in the presence of coupling abrasion and histiocytic reaction, alumina particles do not induce activation of macrophages—as demonstrated by the absence of osteolysis even when there is massive wear.

An additional advantage of alumina-on-alumina coupling is its high corrosion resistance (Nevelos et al. 1999). We measured the serum ion content of stable ceramic-on-ceramic implants and did not find any substantial increase in the release of ions, in contrast to the situation with metal-on-metal couplings (Savarino et al. 2006). Thus, no systemic toxic effects should be expected.

Finally, subclinical infection with slowly growing bacteria might contribute to loosening in cases with uncertain diagnosis.

In conclusion, periprosthetic osteolysis with alumina-on-alumina couplings can be considered to be an effect of the loosening, which is usually due to primary malpositioning and mechanical instability, or trauma or infection, rather than being the cause—which occurs with metal-on-PE bearings, where PE debris has consistently been associated with severe inflammatory reaction and a massive periprosthetic osteolysis. Correct positioning of the implanted prosthesis is crucial in order to avoid complications related to impingement between the femoral neck and the rim of the implant—such as massive abrasion of the ceramic components or femoral head fracture—to achieve low volumes of wear with ceramic-on-ceramic prostheses. If these conditions are fulfilled, the ceramic-on-ceramic bearing may be considered a good choice, particularly for younger patients (Bozic 2005).
